# Current News

**Published:** 1899-08

**Authors:** 


### Current News.

### TO THE ALUMNI OF THE DENTAL DEPARTMENT OF THE UNIVERSITY OF MARYLAND.

With the consent of the Faculty, it is thought fitting that some recognition be made of the founders of scientific thought and dental education in this country.

A movement has been inaugurated within this institution which appeals to every member of the dental profession, but more particularly to our alumni.

It is proposed to erect in the University a memorial tablet to Dr. Horace H. Hayden and Dr. Chapin A. Harris, who are now justly accorded their position as the fathers of dental science.

An elaborate design for a mural tablet, by Mr. Ernest W. Keyser, the American sculptor, embraces alto relievo busts of Drs. Hayden and Harris, which were modelled in Paris from photographs furnished by their respective families. The portraiture of these life-size busts is remarkably faithful, and the design has been accepted by the committee. Blanks for subscription are enclosed, and it is hoped that each dental alumnus will co-operate by even the slightest contribution.

Is it not a happy thought that the Alumni of the School which was the first to encourage scientific dental instruction (dental lectures to medical students given by Dr. Hayden in this University, 1837) should be the first to place in its time-honored halls a memorial tablet, not only to Dr. Hayden, but also to his brilliant collaborator, Dr. Chapin A. Harris?

This statement is made to the Alumni, trusting to their liberality that the unquestioned services rendered by Drs. Hayden and Harris should receive its meed of recognition, and this tardy testimonial reach a successful consummation.

John C. Uhler, M.D., D.D.S.
Isaac H. Davis, M.D., D.D.S.
Clarence J. Grieves, D.D.S.

### HARVARD DENTAL ALUMNI ASSOCIATION.

The third annual "Alumni Day" of the Harvard Dental Alumni Association was observed at the Harvard Dental School on Monday, June 26, 1899, when nearly one hundred and fifty members and friends registered their names with the committee in charge.

The exercises consisted in a display of models and specimens, with patients present, showing the year's work by freshmen, junior, and senior students, embracing all classes in all departments. Papers were read and a symposium given on three subjects; also clinics given on four subjects.

The twenty-eighth annual banquet was observed in the evening at Young's Hotel, Boston, with one hundred and seven individuals seated.

Rev. George C. Lorimer, D.D., pastor of Tremont Temple Church, delivered the annual address, his topic being "The Making of American Character," which he outlined from the race beginning before this country was settled to the present time.

Professor Eugene W. Smith, Dean of the school, the next speaker, gave an outline of the contemplated consolidation of the faculties of the dental, medical, and veterinary schools, with a hospital in connection with these schools.

Professor Thomas Fillebrown gave a *résumé* of the thirty years' advance made by the school since he was a member of the first class to graduate.

Dr. Frederick A. Stevenson, '88, of Montreal, P. Q., addressed the Association, and Herbert A. Reed, of North Attleboro', Mass., responded for the class of '99.

Officers elected for the ensuing year were: President, Edwin C. Blaisdell, '83, Portsmouth, N. H.; Vice-President, Cecil P. Wilson, '72, Boston, Mass.; Secretary, Waldo E. Boardman, '86, Boston, Mass.; Treasurer, Harry S. Parsons, '92, Boston, Mass.

*Executive Committee.*—Waldo E. Boardman, '86, *ex-officio* chairman; William P. Cooke, '81, and Patrick W. Moriarty, '89, all of Boston, Mass.

The Council is composed of the officers of the Association.

WALDO E. BOARDMAN, '86,
*Secretary.*

BOSTON, June 30, 1899.

